# Reconstructing grid emission intensity for Poland and testing its response to EU ETS carbon prices: A replicable two-source triangulation protocol

**DOI:** 10.1016/j.mex.2026.104103

**Published:** 2026-08-18

**Authors:** Adam Dominiak, Artur Rusowicz

**Affiliations:** Warsaw University of Technology, Institute of Heat Engineering, Faculty of Power and Aeronautical Engineering, Nowowiejska 21/25, Warsaw, 00-665, Poland

**Keywords:** Grid emission intensity, Two-source triangulation, Granger causality, Bootstrap inference, EU ETS, Carbon price transmission, Scope 2 emissions, LMDI decomposition

## Abstract

Extended LMDI decomposition incorporating Scope 2 (indirect) emissions requires a reliable time series of grid emission intensity (EFᵍʳⁱᵈ). In Poland, official values are available only from 2014 (KOBiZE), creating a nine-year data gap. This method article documents (i) a fully replicable two-source triangulation protocol reconstructing EFᵍʳⁱᵈ for 2005–2013 from open Eurostat and UNFCCC data, and (ii) a formal test of the relationship between EFᵍʳⁱᵈ and EU ETS allowance (EUA) prices over the single-instrument Phase II–IV window (2008–2022). All inputs derive from open sources with cell-level provenance; every result is reproducible from the deposited data and scripts. EFᵍʳⁱᵈ reconstructed (2005–2013) via two independent paths — a Eurostat fuel-input backcast (R̅ = 0.876; CV = 0.86%) and a UNFCCC CRT allocation (α = 0.856) — which agree to within 9.5 g CO₂/kWh in every year; leave-one-out max error ±13 g CO₂/kWh (1.4%); series spans 928→685 g CO₂/kWh (2005–2022), a 26% decline. Over Phase II–IV (2008–2022), EUA prices Granger-cause grid emission intensity (bootstrap *p* = 0.005 at lag 1, *N* = 5000): a 10% EUA increase is associated with a 0.28% decline in EFᵍʳⁱᵈ the following year (R² = 0.56), consistent with merit-order fuel switching. The result is robust across three inference methods, leave-one-out year exclusion, and all reconstruction variants. No reverse causality is detected (CO₂,ind ↛ EUA, *p* > 0.62) and transmission to industrial emissions is weak (*p* = 0.078). EFᵍʳⁱᵈ should therefore be treated as predetermined but not strictly exogenous in LMDI and panel applications; the decomposed grid effect partially embeds a carbon-price response, which we quantify.

## Graphical abstract

**Figure:**
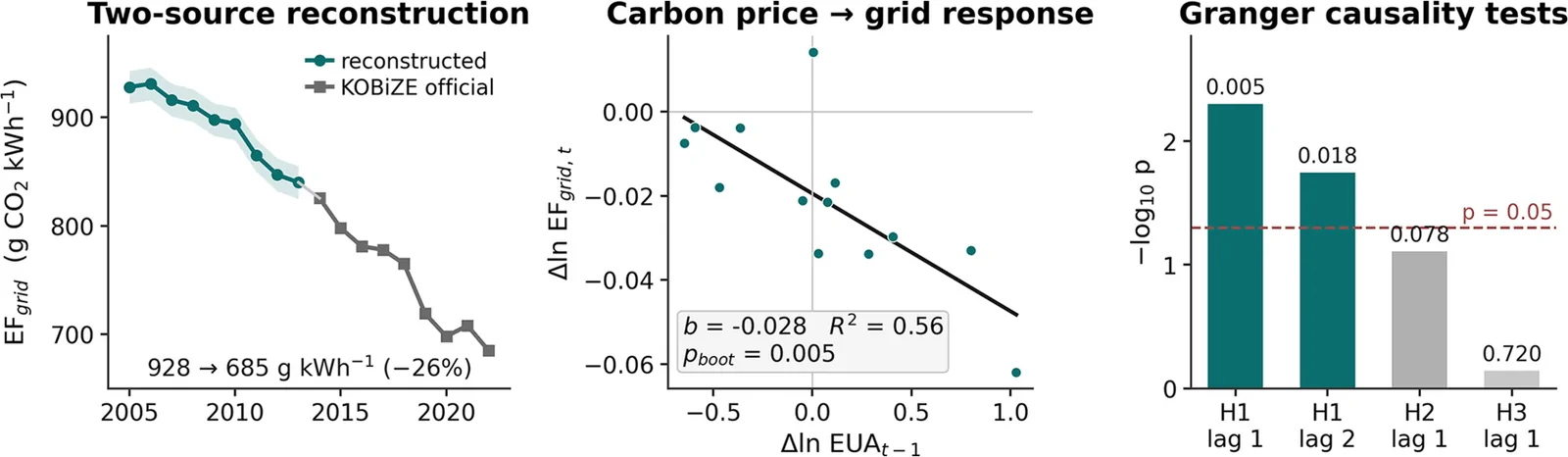

## Specifications table

*This table provides general information on your method.*

**Table:** 

**Subject area**	Energy economics; Environmental science; Statistical methods
**More specific subject area**	Grid emission intensity; Carbon price transmission; LMDI decomposition; Granger causality; Bootstrap inference; EU ETS
**Name of your method**	Two-source triangulation for EFᵍʳⁱᵈ reconstruction with bootstrap Granger testing of carbon-price response
**Name and reference of original method**	LMDI: Ang [[1], [2], [3]]. Granger causality: Granger [4]; Toda–Yamamoto [5]; Hacker–Hatemi-J bootstrap [6]. VAR: Sims [7]. Scope 2 accounting: GHG Protocol [8]
**Resource availability**	All data open access: Eurostat nrg_bal_c, nrg_cb_e, env_ac_ainah_r2 (https://ec.europa.eu/eurostat); UNFCCC CRT Poland, submission POL-CRT-2024-V0.1 (https://unfccc.int); KOBiZE annual reports (https://www.kobize.pl); ICAP Allowance Price Explorer, secondary market (https://allowancepriceexplorer.icapcarbonaction.com). Complete replication package (data, R scripts, provenance): Zenodo, 10.5281/zenodo.21647724.

## Background

Extended Log-Mean Divisia Index (LMDI) decomposition of industrial CO₂ emissions incorporating Scope 2 (indirect) emissions requires a continuous, internally consistent time series of grid emission intensity (EFᵍʳⁱᵈ) for the full study period. Scope 2 emissions arise from purchased electricity and are allocated to industrial sectors using EFᵍʳⁱᵈ [8]. Without a complete series, decomposition must either truncate the analysis or rely on unjustified extrapolation.

In Poland, KOBiZE has published annual end-consumer EFᵍʳⁱᵈ values only from 2014 onwards [9]. Study periods relevant to EU ETS policy evaluation typically begin in 2005 or 2008, creating a data gap of up to nine years for which no official Polish series exists.

A second methodological question concerns the relationship between EFᵍʳⁱᵈ and the EU ETS allowance (EUA) price. If the carbon price systematically drives grid decarbonisation, the decomposed grid effect in LMDI partially reflects a policy response rather than an autonomous technology trend, and panel specifications using contemporaneous EFᵍʳⁱᵈ face endogeneity concerns. Whether such a relationship exists in a coal-dominated system is an empirical question that requires formal testing rather than assertion in either direction.

This article addresses both problems. First, it documents a two-source triangulation protocol that reconstructs EFᵍʳⁱᵈ for 2005–2013 by exploiting stable structural relationships between independently derived Eurostat and UNFCCC data. Second, it tests Granger causality between EUA prices, EFᵍʳⁱᵈ, and sectoral industrial CO₂ emissions over the Phase II–IV window (2008–2022), with small-sample bootstrap inference as the primary method. The protocol is transferable to other Central and Eastern European countries after country-specific recalibration.

## Method details

**1. Data gap and rejection of interpolation**

Linear interpolation was rejected because the Polish generation mix underwent non-linear structural change between 2005 and 2013: cyclical coal utilisation around the 2008–2009 crisis and rapid wind deployment after 2010 [10,11]. Two-source triangulation exploits stable structural relationships instead of imposing a functional form. All values are location-based, consistent with physical electricity flows in the Polish system [8].

**2. Method 1 — Eurostat fuel-input backcast**

The implicit fossil-fuel emission intensity EFᴱᵘʳ(t) is computed from Eurostat energy balances (nrg_bal_c, transformation input code TI_EHG_E) for nine SIEC fuel categories with IPCC 2006 default emission factors (t CO₂/TJ): coking coal and other bituminous coal 94.6, lignite 101.0, coke-oven gas 44.4, blast-furnace gas 260.0, natural gas 56.1, refinery gas 57.6, gas/diesel oil 74.1, fuel oil 77.4. The denominator is gross electricity production (nrg_cb_e, code GEP). For the nine overlap years 2014–2022 the ratio Rₜ = EFᵍʳⁱᵈ,KOBiZE(t) / EFᴱᵘʳ(t) is highly stable: R̅ = 0.876, σ = 0.008, CV = 0.86%. Leave-one-out cross-validation yields a maximum prediction error of ±13.2 g CO₂/kWh (1.4%). Backcast: EFᴮᵃᶜᵏ(t) = EFᴱᵘʳ(t) × R̅ for t ∈ {2005,…,2013}.

Two structural factors explain R̅ < 1: transmission-and-distribution losses (the KOBiZE factor is an end-consumer quantity, lower by ∼7–9% [12]) and the reference-efficiency CHP allocation applied by KOBiZE (GHG Protocol [8]; Directive 2012/27/EU Annex II [13]) versus the IPCC Tier 1 implicit factor. Both are slow-moving system properties, which is why CV is below 1%; intra-window stability is confirmed by a first-half/second-half split of the overlap period (R̅ difference < 0.01).

**3. Method 2 — UNFCCC CRT allocation**

An allocation coefficient α apportions the joint electricity-and-heat emission total of UNFCCC Common Reporting Tables (CRT; formerly CRF) category 1.A.1.a to the electricity stream [14]. We use the Poland submission POL-CRT-2024-V0.1 (26 November 2024), worksheet Table1.A(a)s1, with cell-level provenance for every value. Calibration on 2014 — the first overlap year with the official series — yields α = 0.856. The estimate is EFᶜʳᵗ(t) = CRTₜ × α × 10⁶ / P(t), with P(t) gross generation. The exceedance of α over a proportional energy-output split [15] reflects the reference-efficiency method.

**4. Triangulation and uncertainty**

**Table 1:** EF^grid^ series for Poland, 2005–2022 (g CO₂/kWh). Reconstructed values: triangulation of two independent paths; official KOBiZE values from 2014. Path divergence = |Backcast − CRT|.

Year	EF^grid^	Method	Backcast	CRT path	|Δ|
2005	928	Triangulation	926.6	929.0	2.4
2006	931	Triangulation	930.8	931.9	1.1
2007	916	Triangulation	915.9	916.2	0.3
2008	911	Triangulation	910.9	911.5	0.6
2009	898	Triangulation	897.2	898.3	1.1
2010	894	Triangulation	892.9	895.9	3.0
2011	865	Triangulation	861.1	868.5	7.4
2012	847	Triangulation	842.5	851.0	8.5
2013	840	Triangulation	835.7	845.2	9.5
2014	825.412	KOBiZE official	—	—	—
2015	798	KOBiZE official	—	—	—
2016	781	KOBiZE official	—	—	—
2017	778	KOBiZE official	—	—	—
2018	765	KOBiZE official	—	—	—
2019	719	KOBiZE official	—	—	—
2020	698	KOBiZE official	—	—	—
2021	708	KOBiZE official	—	—	—
2022	685	KOBiZE official	—	—	—

**Fig. 1:**
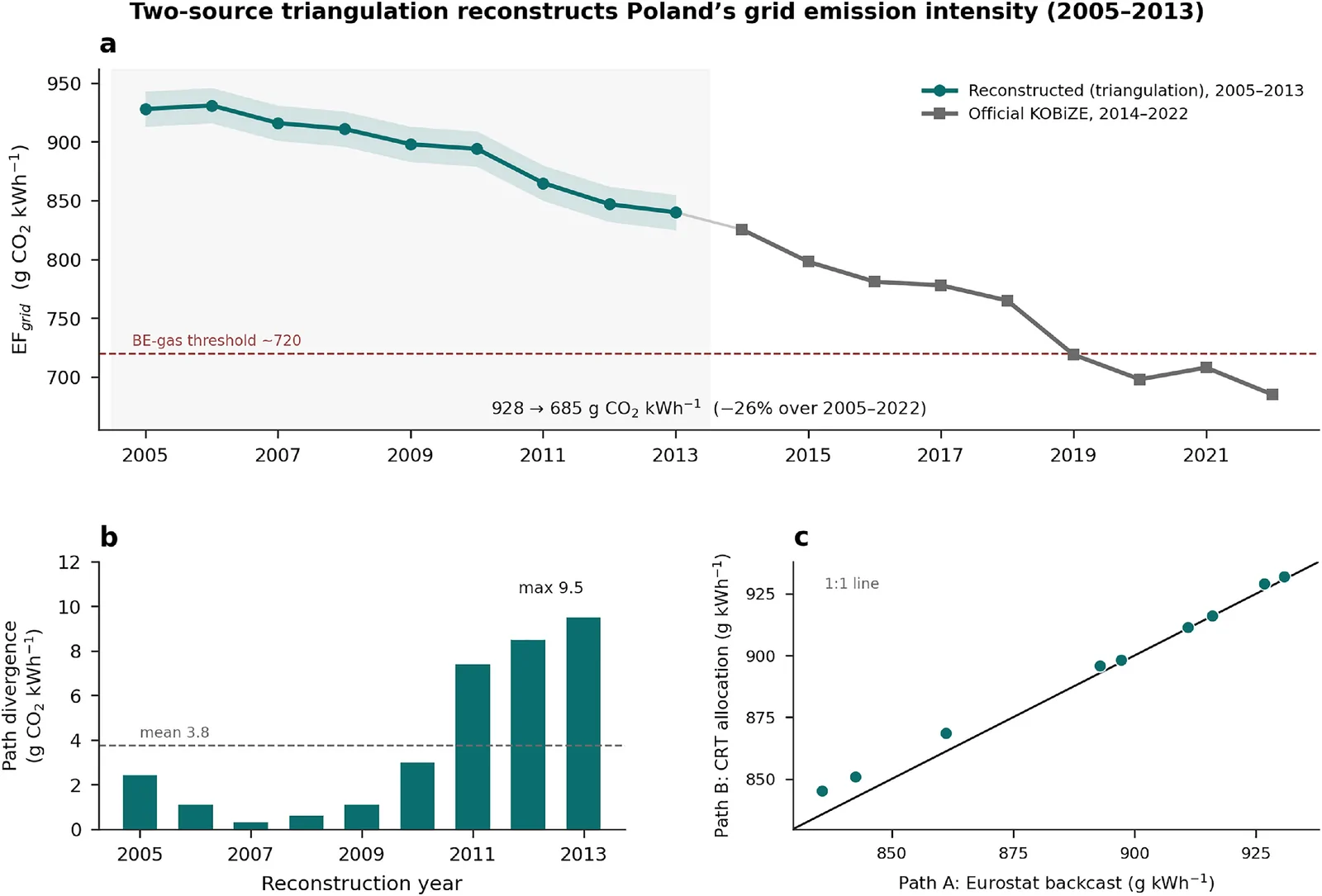
Two-source triangulation of Poland’s grid emission intensity. (a) Full series with reconstruction uncertainty band and BE-gas threshold; (b) year-by-year path divergence (max 9.5 g/kWh); (c) agreement of the two independent paths against the 1:1 line.

The final estimate is the arithmetic mean of the two paths. With the refined fuel-input recipe the paths agree to within 9.5 g CO₂/kWh in every reconstructed year (mean divergence 3.8 g), versus ±21 g in an earlier coarser specification — direct evidence against shared bias, since the two paths rest on entirely different primary sources and error structures. Monte Carlo propagation (*N* = 10,000, σ = 7 g/kWh) yields a 90% band of ±0.54% on the cumulative reconstructed series. Reconstructed values (g CO₂/kWh): 927.8 (2005), 931.3, 916.1, 911.2, 897.7, 894.4, 864.8, 846.7, 840.4 (2013); official KOBiZE values are used unchanged from 2014 (Table 1, Fig. 1).

**5. Estimation window and series for causality testing**

The causality analysis uses 2008–2022 (T = 15 annual observations; 14 first differences), chosen on two grounds. First, a single allowance instrument applies throughout: Phase I EUAs (2005–2007) were non-bankable into Phase II and collapsed to €0.86 by 2007, constituting a different asset whose inclusion contaminates Δln(EUA). Second, every input is reproducible from currently available public sources: sectoral industrial CO₂ (env_ac_ainah_r2, NACE C10–C12, C17, C20, C23, C24) begins in 2008 with the introduction of NACE Rev. 2. EUA prices are daily secondary-market quotations from the ICAP Allowance Price Explorer [16] (5,349 observations), averaged annually; the 2008 mean uses Phase II observations only (April–December, *n* = 190), as the series tracks the expiring Phase I contract until March 2008. EFᵍʳⁱᵈ is the Table 1 series.

**6. Unit roots, cointegration, and specification**

ADF and KPSS tests (Table 3) indicate that ln(EFᵍʳⁱᵈ) is I(1) and stationary after differencing; ln(CO₂,ind) is borderline trend-stationary; ln(EUA) shows high persistence with mixed evidence even in differences — a known feature of carbon-price series at annual frequency. The Johansen trace test suggests up to two cointegrating relations, but at T = 15 it has negligible power and is reported only descriptively. Given the uncertain integration orders, inference proceeds on three complementary tracks: (i) asymptotic Granger F-tests on Δln series; (ii) the Hacker–Hatemi-J residual bootstrap (*N* = 5,000) [6], robust to small samples and non-normality, taken as the primary method; and (iii) the Toda–Yamamoto lag-augmented Wald test on levels [5], valid regardless of integration and cointegration. AIC and BIC select lag order 2 for the differenced system; Granger tests are reported for lags 1–3 (lag 4+ is infeasible at this T). VAR(1) diagnostics: stable roots, no residual autocorrelation (Portmanteau *p* = 0.38), normality not rejected (Jarque–Bera *p* = 0.85). Full test results are reported in Table 2.

**7. Granger causality results**

**Table 3:** Unit root and stationarity tests (2008–2022). ADF H₀: unit root; KPSS H₀: stationarity. Mixed evidence for ln EUA motivates the three-track inference strategy.

Series	ADF level (p)	ADF Δln (p)	KPSS level (p)	KPSS Δln (p)
ln EUA	−0.29 (0.926)	−0.78 (0.824)	0.237 (0.10)	0.504 (0.04)
ln EF^grid^	2.59 (0.999)	−7.54 (0.000)	0.599 (0.02)	0.173 (0.10)
ln CO2,ind	−3.32 (0.014)	−5.61 (0.000)	0.139 (0.10)	0.130 (0.10)

**Fig. 2:**
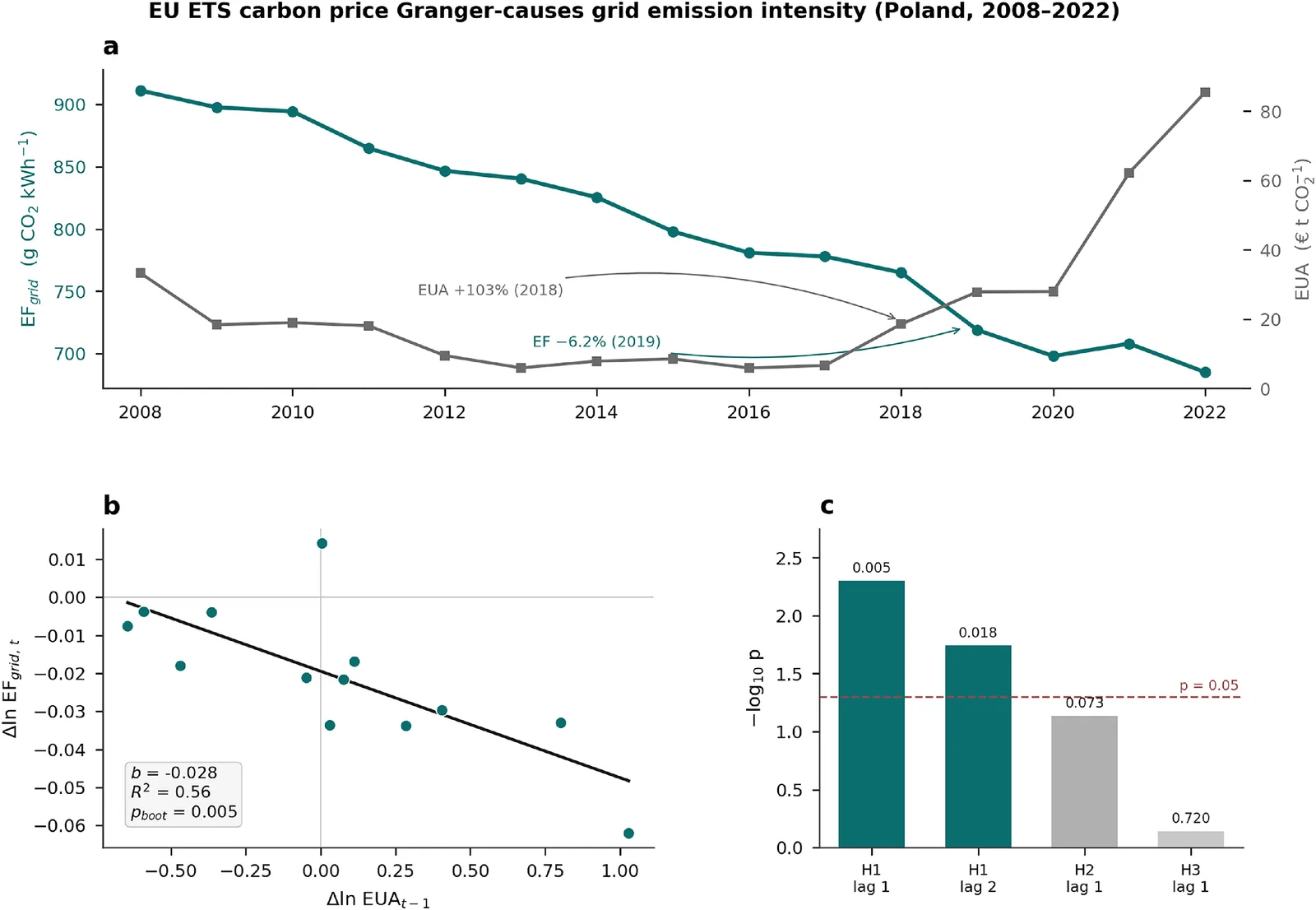
Carbon-price response of grid emission intensity, Poland 2008–2022. (a) EF^grid^ and EUA price trajectories; (b) Δln EUA(t − 1) vs Δln EF^grid^(t) with OLS fit (b = −0.028, R² = 0.56); (c) −log₁₀ p across hypotheses and lags with the 5% threshold.

Table 2 reports all three hypotheses across lags 1–3 and all three inference methods. H1: EUA prices Granger-cause EFᵍʳⁱᵈ — bootstrap *p* = 0.005 (lag 1) and 0.041 (lag 2); Toda–Yamamoto Wald *p* < 0.001 (p_opt = 1). The effect size from ΔlnEFₜ = a + b·ΔlnEUAₜ₋₁ is b = −0.028 (HC1 SE 0.007, R² = 0.56): a 10% EUA increase is associated with a 0.28% decline in grid intensity the following year. The 2018→2019 episode illustrates the channel: a + 103% EUA jump preceded a −6.2% EFᵍʳⁱᵈ drop (predicted: −4.8%). The one-year lag and negative sign are consistent with merit-order fuel switching, by which higher carbon prices raise the marginal cost of coal relative to gas and renewables [17,18]. H2: transmission to sectoral industrial CO₂ is weak (bootstrap *p* = 0.078 at lag 1 only) and treated as indicative at most. H3: no reverse causality from Polish industrial emissions to the EUA price (p > 0.62 at all lags), consistent with price-taking in an EU-wide market [19]. Robustness: H1 significance at the 1% level (lag 1) holds for all three EFᵍʳⁱᵈ variants (backcast-only, CRT-only, triangulated), under leave-one-out exclusion of every sample year (max *p* = 0.094), and across both independent implementations (R and Python, identical statistics to three decimals) (Fig. 2).

**8. Implications for decomposition and Scope 2 practice**

**Table 2:** Granger causality tests, Poland 2008–2022: three hypotheses × lags 1–3 × three inference methods. Bootstrap: Hacker–Hatemi-J, *N* = 5,000. TY: Toda–Yamamoto MWALD on levels (p_opt = 1).

Hypothesis	Lag	F	p (asympt.)	p (bootstrap)	p (TY levels)
H1: EUA → EF^grid^	1	12.87	0.005	0.005	< 0.001
	2	7.60	0.018	0.041	—
	3	4.90	0.079	0.135	—
H2: EUA → CO₂,ind	1	4.01	0.073	0.078	0.375
	2	1.67	0.255	0.262	—
	3	0.70	0.601	0.594	—
H3: CO₂,ind → EUA	1	0.14	0.720	0.717	0.749
	2	0.45	0.653	0.648	—
	3	0.02	0.995	0.995	—

Three practical consequences follow. First, LMDI decomposition itself remains fully valid: it is an accounting identity and does not require exogeneity of EFᵍʳⁱᵈ. However, the decomposed grid-emission effect should be interpreted as partially embedding a carbon-price response rather than an autonomous technology trend; the coefficient above quantifies that component. Second, panel specifications should prefer lagged EFᵍʳⁱᵈ as a predetermined regressor; contemporaneous use requires instrumenting. Third, the choice of Location-based Scope 2 accounting rests on structural grounds — in CEE markets with over 88% of consumption untracked by Guarantees of Origin [20], the Market-based method is uninformative — and does not depend on the causality result. These recommendations transfer to other CEE systems after recalibration of R̅ and α on country-specific overlap windows.

## Method validation

### Validation proceeds at five levels.

**Cross-source agreement.** The two reconstruction paths, resting on disjoint primary sources, agree to within 9.5 g CO₂/kWh in every year (mean 3.8 g; Fig. 1b, c). Shared bias would require the same error to arise independently in Eurostat energy balances and the national UNFCCC inventory.

**Leave-one-out cross-validation.** Maximum prediction error across the nine overlap folds is ±13.2 g CO₂/kWh (1.4%); the stable-ratio assumption is not sensitive to any single calibration year, and the first-half/second-half ratio split confirms intra-window stability.

**Monte Carlo propagation.** *N* = 10,000 draws (σ = 7 g/kWh per reconstructed year) give a 90% band of ±0.54% on the cumulative reconstructed series.

**Inference triangulation.** Three methods with different validity conditions — asymptotic F on differences, residual bootstrap, and lag-augmented Wald on levels — yield the same qualitative pattern for all hypotheses (Table 2), and two independent implementations (R, Python) reproduce identical deterministic statistics.

**Configuration transparency.** A full configuration decomposition (Supplementary Table S2) shows how the H1 conclusion depends on the estimation window: on 2005–2022 including Phase I the predictive signal is masked (p > 0.20), while any configuration confined to Phase II–IV — with either the earlier or the corrected data — detects it (p ≤ 0.01). The reversal is therefore attributable to the exclusion of the non-bankable Phase I contract, not to data replacement.

## Limitations

Sample size. T = 14 first differences is small; asymptotic Granger tests over-reject in near-integrated systems [21]. The bootstrap is therefore the primary inference method, and all conclusions are framed as predictive Granger causality — indicative evidence, not definitive causal identification. Controls for confounders (fuel prices, demand, renewable policy) are infeasible at this dimensionality.

Annual frequency. The merit-order mechanism operates at hourly resolution; annual aggregation conflates dispatch-order effects with slower investment responses. The one-year lag structure should be read accordingly.

Bivariate tests. Trivariate systems with multiple lags are infeasible at this sample size; omitted-variable bias relative to a full structural model cannot be excluded.

Window exclusions. Results say nothing about Phase I (2005–2007), excluded by design as a different, non-bankable instrument.

Single-country calibration. R̅ and α are Poland-specific. Transfer to other CEE systems requires recalibration on country overlap windows; for countries whose official series begins later, shorter windows will widen the LOO error.

## Ethics statements

This study uses only publicly available administrative and statistical data (Eurostat, UNFCCC, KOBiZE, ICAP). No human subjects, animal experiments, or social media data were involved. No ethics approval was required.

## Related research article

None.

## CRediT authorship contribution statement

**Adam Dominiak:** Conceptualization, Data curation, Formal analysis, Methodology, Visualization, Writing – original draft. **Artur Rusowicz:** Supervision, Validation, Writing – review & editing.

## Declaration of competing interest

The authors declare that they have no known competing financial interests or personal relationships that could have appeared to influence the work reported in this paper.

## Data Availability

Replication package (data, code, provenance): 10.5281/zenodo.21647724.

## Appendix Supplementary materials

Supplementary material *and/or* additional information [OPTIONAL] Supplementary Table S1: full reconstruction worksheet (all source values, ratios, both paths, divergences). Supplementary Table S2: configuration decomposition of the Granger results across estimation windows and data vintages. Replication package (Zenodo, 10.5281/zenodo.21647724): all raw data with provenance (9 KOBiZE reports, CRT extraction with cell addresses, Eurostat API pulls, 5349 ICAP daily prices), processed series, and R scripts 01–04 reproducing every table and figure; Python cross-check scripts included. Data availability: all sources open access as listed in the Specifications table; no commercial data are used. Image, application 1
